# Electrocardiographic Changes in a Horse with Induced Myocardial Infarction

**DOI:** 10.3390/ani12101272

**Published:** 2022-05-16

**Authors:** Rikke Weis, Helena Carstensen, Stefan M. Sattler, Rikke Buhl, Eva M. Hesselkilde

**Affiliations:** 1 Department of Veterinary Clinical Sciences, Faculty of Health and Medical Sciences, University of Copenhagen, Højbakkegaard Allé 5, 2630 Taastrup, Denmark; rps816@alumni.ku.dk (R.W.); hc@sund.ku.dk (H.C.); evah@sund.ku.dk (E.M.H.); 2 Department of Cardiology, Gentofte University Hospital, Gentofte Hospitalsvej 1, 2900 Hellerup, Denmark; sattler@sund.ku.dk; 3 Department of Biomedical Sciences, Faculty of Health and Medical Sciences, University of Copenhagen, 2200 Copenhagen, Denmark

**Keywords:** equine cardiology, Copenhagen method, 12-lead ECG, myocardial infarct, acute myocardial infarction, horse

## Abstract

**Simple Summary:**

During acute myocardial infarction (AMI), the electrophysiological changes are clearly visible on the 12-lead electrocardiogram (ECG), and are as such, an important diagnostic tool in human cardiology. Similar to humans, changes are seen on the ECG in both pigs and dogs, but so far, this has not been studied in horses, despite equine cardiology being a growing field. This study aimed to investigate the ECG changes seen with a 12-lead ECG in a horse with induced myocardial infarction. The ECG changes observed in this case report were comparable to other species with AMI and showed similar patterns throughout the different phases of occlusion. The results could, therefore, indicate that a 12-lead ECG can be used to detect signs of ischemic heart disease, such as AMI, in horses, while they also potentially elucidate certain translational connections between human and veterinary medicine.

**Abstract:**

During acute myocardial infarction (AMI), the ischemia and necrosis of the infarcted tissue result in local electrophysiological changes, which bring about deviations of the ST segment and T wave. In this case report, the aim was to investigate whether these changes could be detected with a 12-lead electrocardiogram (ECG) during acute occlusion of the coronary artery in a 15-year-old Standardbred mare (scheduled for euthanasia due to non-cardiac health problems). The left anterior descending (LAD) coronary artery was occluded using an angioplasty balloon catheter guided through the carotid artery. Two coronary occlusions of 30 min were induced, separated by a 10-min reperfusion phase. AMI led to ST deviations and T-wave amplitude changes (maximum ST deviation was 1.98 mV; T-wave amplitude increased from 6.58 to 9.25 mV). The ST segment almost returned to the baseline during the reperfusion phase. The ECG changes seen after the infarction were comparable to those reported in other species with AMI, suggesting that the 12-lead-ECG can potentially be used to detect signs of myocardial infarction in horses.

## 1. Introduction

In human medicine, the 12-lead electrocardiogram (ECG) is an important diagnostic tool for the detection of arrhythmias, cardiac hypertrophy and myocardial infarction (MI) [1,2]. Acute myocardial infarction (AMI) occurs when a coronary artery is occluded, e.g., due to plaque rupture, erosion or dissection, which leads to downstream ischemia and necrosis of the cardiac tissue. The subsequent changes in the electrophysiological properties of the heart can, in humans and other animals such as sheep, primates, dogs and pigs, be recorded on the ECG [3]. Acute transmural ischemia will result in a rapid-onset increase of the T-wave amplitude, followed by the ST-segment elevation in adjacent ECG leads, while leads facing away can show no to minor changes or ST-segment depression. In humans, this makes the 12-lead ECG the cornerstone in diagnosing transmural AMI, termed ST-elevation myocardial infarction (STEMI), where changes threaten within minutes. The anatomical positions of ECG leads with ST elevation, in relation to nearby coronary vessels, are used to identify the culprit lesion [4].

In equine medicine, ECG recordings are generally used to detect arrhythmias, and often only one to three leads is used [5]. Recently, a new method for using 12-lead ECG in horses was described to obtain more detailed information on the equine heart [6]. However, to our knowledge, no one has yet studied whether transmural ischemia can be diagnosed on equine ECGs. In that context, we hypothesized that the 12-lead ECG can be used in horses to detect myocardial ischemia induced by the occlusion of a coronary artery.

## 2. Materials and Methods

### 2.1. Acute Myocardial Infarction

**Figure 1:**
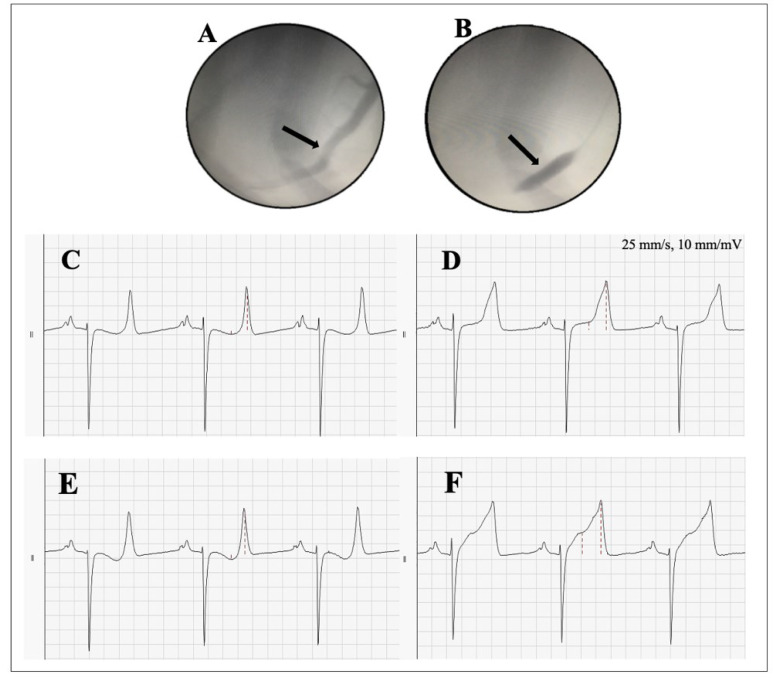
Occlusion of the left anterior descending (LAD) coronary artery and the corresponding electrocardiogram (ECG). (**A**) Angiogram of the LAD (arrow), (**B**) Showing the balloon catheter positioned distally in the LAD (arrow), (**C**) Lead II two minutes before occlusion, (**D**) Lead II two minutes after the first occlusion, (**E**) Lead II five minutes after reperfusion, (**F**) Lead II two minutes after the second occlusion. In figure (**C**–**F**) the red dotted lines illustrate the points of measurements.

A 15-year-old Standardbred mare was scheduled for euthanasia (due to non-cardiac health problems) and included in this study. Before including this horse in the study, a full clinical examination, blood sample and resting ECG were performed. The horse was sedated and anesthetized as previously described and placed in dorsal recumbency [7]; the horse did not recover from anesthesia. Under general anesthesia, a cutdown approach was used to access the left carotid artery, and a 16 F sheath (30 cm, Check-Flo Performer Introducer, William Cook Europe ApS, 4632 Bjæverskov, Denmark) was inserted. A 13 F delivery system (TorqVue 45, AGA Medical Corporation, Plymouth, MN, USA) was advanced over a guidewire (0.035″ angled, Radiofocus, Terumo Medical Corporation, Tokyo, Japan) to the ostium of the left coronary artery. The left anterior descending (LAD) coronary artery was visualized by angiogram using contrast fluid (Visipaque 320 mg/mL, GE Healthcare A/S, Brøndby, Denmark) and fluoroscopy (Siremobil Compact C-Arm, Siemens, Munich, Germany). The guidewire was directed into the LAD and a 10 × 38 mm angioplasty balloon (Advanta V12, Atrium Medical, Merrimack, NH, USA) was placed over the wire and positioned in the LAD (Figure 1A,B). For this study, two occlusions were performed. At first, the balloon was positioned in the medial part of the LAD and inflated for 30 min followed by deflation and subsequent 10-min reperfusion. The angioplasty balloon was then retracted by approximately 4 cm into a more proximal part of the LAD and inflated, which resulted in a larger ischemic area.

### 2.2. ECG Recording and Measurements

**Figure 2:**
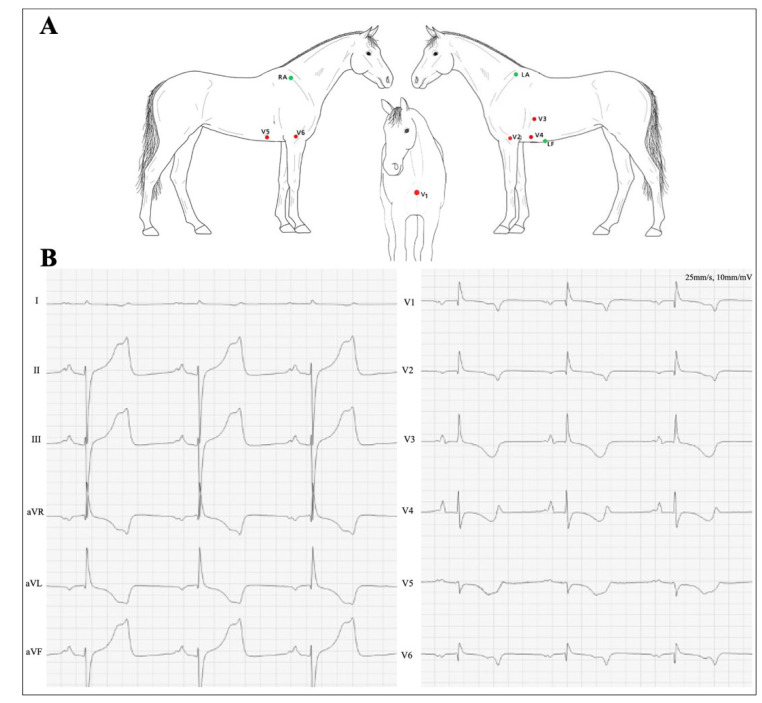
(**A**) Copenhagen Method electrode placement, figure reprinted with permission from Hesselkilde et al 2020 JVIM [6]. (**B**) All leads seen two minutes after the second occlusion. LA: left arm, RA: right arm, LF: left foot, I: Lead I, II: Lead II, III: Lead III, Augmented vector right: aVR, Augmented vector left: aVL, Augmented vector foot: aVF. Precordial leads: V1–V6.

During the procedure, a 12-lead ECG (GE Marquette CAM-14, GE Healthcare A/S, Brøndby, Denmark), with the electrodes placed according to the recently published 12-lead method, the “Copenhagen method” [6] (Figure 2A), was recorded and analyzed off-line using the associated software (Cardiosoft, GE Healthcare A/S, Brøndby, Denmark). Leads II (RA—LF) and III (LA—LF) in the Copenhagen Method resemble the “modified base-apex” ECG most often used in clinical practice. The ST-segment amplitude (measured 200 ms after the J-point) (Figure 1C–F) and T-wave amplitude were measured in all 12 leads for three consecutive beats at four different time points: baseline, 2 min after the first occlusion, 5 min after reperfusion and 2 min after the second occlusion. Data are presented as the sum of the absolute changes in all 12 leads:

$$\underset{all\;ECG\;leads}{\sum }abs\;\left(ST\;amplitude\;at\;J\;point\;or\;T\;amplitude\right)$$

## 3. Results

**Figure 3:**
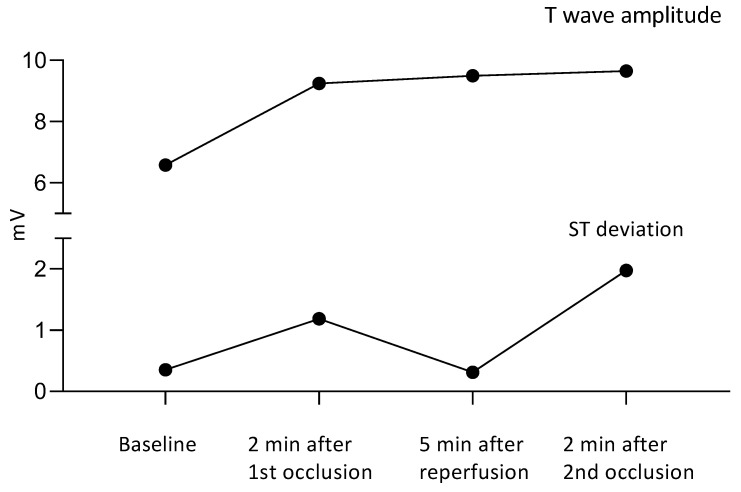
The sum of the ST deviations and T-wave amplitudes.

The sum of ST deviations (STD) at the baseline was 0.35 mV, and two minutes following the first occlusion, clear ST deviations were visible with a sum of 1.19 mV. During reperfusion, the ST deviations returned to the baseline with a sum of 0.31 mV. Two minutes into the second occlusion, an even more pronounced STD of 1.98 mV was observed (Figure 3). The ST-segment and T-wave amplitude changes over time can visually be seen in lead II (Figure 1C–F). The STD was most distinct in leads II, III and aVF and in V3 and V4 in the precordial leads. The ST segment was elevated in leads I, II, III and aVF, while ST-segment depression was seen in leads aVR, aVL and the precordial leads V1–6 (Figure 2B).

The baseline sum of the T-wave amplitude was 6.58 mV, and two minutes into the first occlusion, the sum was 9.25 mV. Five minutes after the reperfusion, the sum of the T-wave amplitude changes was 9.50 mV, and two minutes into the second occlusion, the sum was 9.65 mV (Figure 3).

During the reperfusion phase, after the second occlusion, the horse developed ventricular fibrillation and asystole while under anesthesia. Post-mortem examination of the heart confirmed occlusion of the proximal LAD.

## 4. Discussion

In this study, we showed that acute occlusion of a coronary vessel and subsequent transmural AMI in a horse resulted in ST deviation changes in anatomically related ECG leads.

Research on equine myocardial infarction (MI) is very limited [8], and to our knowledge, no studies have induced MI in horses or monitored an ischemic event with a 12-lead ECG. One study executed LAD occlusions for a couple of minutes by open-heart surgery in six ponies, and followed them for 27 days post-operationally to study their myocardial function, but no ECGs were recorded during the study [9]. Recently, the anatomical site of premature atrial and ventricular depolarizations was described in horses using a 3D mapping system [10,11], and it could be interesting in future studies combining 3D mapping with AMI research in horses to further correlate ECG findings with anatomical mapping.

During transmural AMI, diastolic injury currents and increased extracellular potassium concentrations lead to ST elevation and high T-wave amplitudes [3]. Bipolar leads (I, II, III, aVL, aVR and aVF) facing the ischemic area will present the most prominent changes to the ST segment and T wave. Unipolar leads (V1–6) directly facing the ischemic area will also present ST-segment elevation [4]. Therefore, the positions of ECG leads in relation to the anatomical area with ischemia can be used to diagnose the affected coronary vessel. Yet, due to our limited knowledge of AMI in horses, it is premature to say we can yet adapt the human clinical experience to horses. Furthermore, we used fluoroscopy guidance to place the balloon in the coronary artery, a diagnostic imaging modality that is standard in human cardiology but had not previously been applied in equine cardiology. This technique has not yet been validated in horses, and mainly due to the large size of a horse, may be challenging to apply.

In our study, the ST segment was elevated in leads II, III and aVF, while ST-segment depression was seen in V3 and V4. The positions of the ECG leads when using the Copenhagen method resulted in coverage of the LAD perfused area, mainly by leads II, III and aVF. In humans, these leads face the inferior parts of the heart (diaphragmatic surface mainly formed by the right ventricle) and are largely used in diagnostics for occlusion of the right coronary artery [4].

In horses, the precordial leads are placed around the chest to cover all chambers of the heart. The altered lead positioning, and distance from the electrodes to the heart and thickness of the equine myocardium, may lead to findings other than those expected based on human procedures [6]. In this study, leads V3 and V4 were the closest anatomically to the myocardial tissue supplied by the occluded coronary vessel, and as expected, these two leads were also the precordial leads that showed the greatest deflections. This supported our hypothesis that this particular occlusion leading to myocardial ischemia could be detected using the 12-lead ECG method in this horse.

In humans, the precordial ECG leads are placed close together on the chest to provide good anatomical coverage of the anterior, left-lateral and segmental wall segments (LAD and diagonal branches). The posterior wall and right-sided segments are clearly underrepresented, and transmural AMI in these regions is often only diagnosed indirectly by ST depression in the leads facing away [4].

In human cardiology, an ST-segment change of at least >0.1 mV in two adjacent leads is diagnostic for AMI [4]. In this study, we observed an ST-segment change of 1.62 mV from the baseline until two minutes after the second occlusion, which indicates that the changes seen in this horse were similar to the expected findings in humans. In the studied horse, ST-segment changes nearly resolved to the baseline levels within five minutes of reperfusion, suggesting that 30 min of acute occlusion does not induce permanent tissue damage.

The ST-segment changes and their dynamics were similar to those in other species with induced AMI [12]. The T-wave amplitude did not change as much as the ST segment throughout the study, but the amplitude did increase following the occlusions (Figure 3).

### Limitations

Detection of myocardial damage depends not only on the electrode position but also on factors such as the distance from the damaged tissue, intervening structures and the strength of a wavefront, which will influence the electrocardiographic appearance and are thus considered limitations that impact the utility of the technique. To support the ECG findings and provide a deeper understanding of the myocardial tissue damage, measurements of biomarkers such as troponin could have been taken. Furthermore, a thorough postmortem examination of the horse including the use of histopathology could have provided more valuable information on specific tissue damage in the myocardial area vascularized by the LAD. It is worthy of note that the size of the infarct made in this case study was large, and therefore, was expected to result in different and probably more severe electrocardiographic findings compared to clinical cases. The study setup in this case report did not allow for a possible clinical evaluation of the horse following the AMI.

## 5. Conclusions

The results of this study demonstrate that AMI of the magnitude induced can be detected with a body-surface ECG with the lead placement used here. The difference in the leads suggests that the infarcted area could potentially be located and diagnosed in horses using the 12-lead Copenhagen method for ECG. As the horse did not recover from anesthesia, the long-term effect of myocardial infarction on the horse could not be studied. Since only one horse was included in this case study, whether the ECG changes apply to all horses still needs further investigation with larger study populations.

## Data Availability

Not applicable.
